# Supplementary material for: Predicting visual function by interpreting a neuronal wiring diagram
Source: Nature. 2024 Oct 2;634(8032):113–23. doi: 10.1038/s41586-024-07953-5 (PMC11446822; doi:10.1038/s41586-024-07953-5)
Supplement: Supplementary file 6 — CRF and ERF predictions for individual TmY4 and TmY9 cells. Analogous to Supplementary Data 3, but for TmY target types. Shown are the top four monosynaptic pathways, the strongest pathway passing through each of the top ten intermediary types (ranking from Extended Data Fig. 7), and the trisynaptic pathway Tm1–TmY–Dm3–TmY (see the section entitled Prediction of spatial normalization). [file 41586_2024_7953_MOESM6_ESM.zip › DataS4/TmY9q/720575940631087301.pdf]

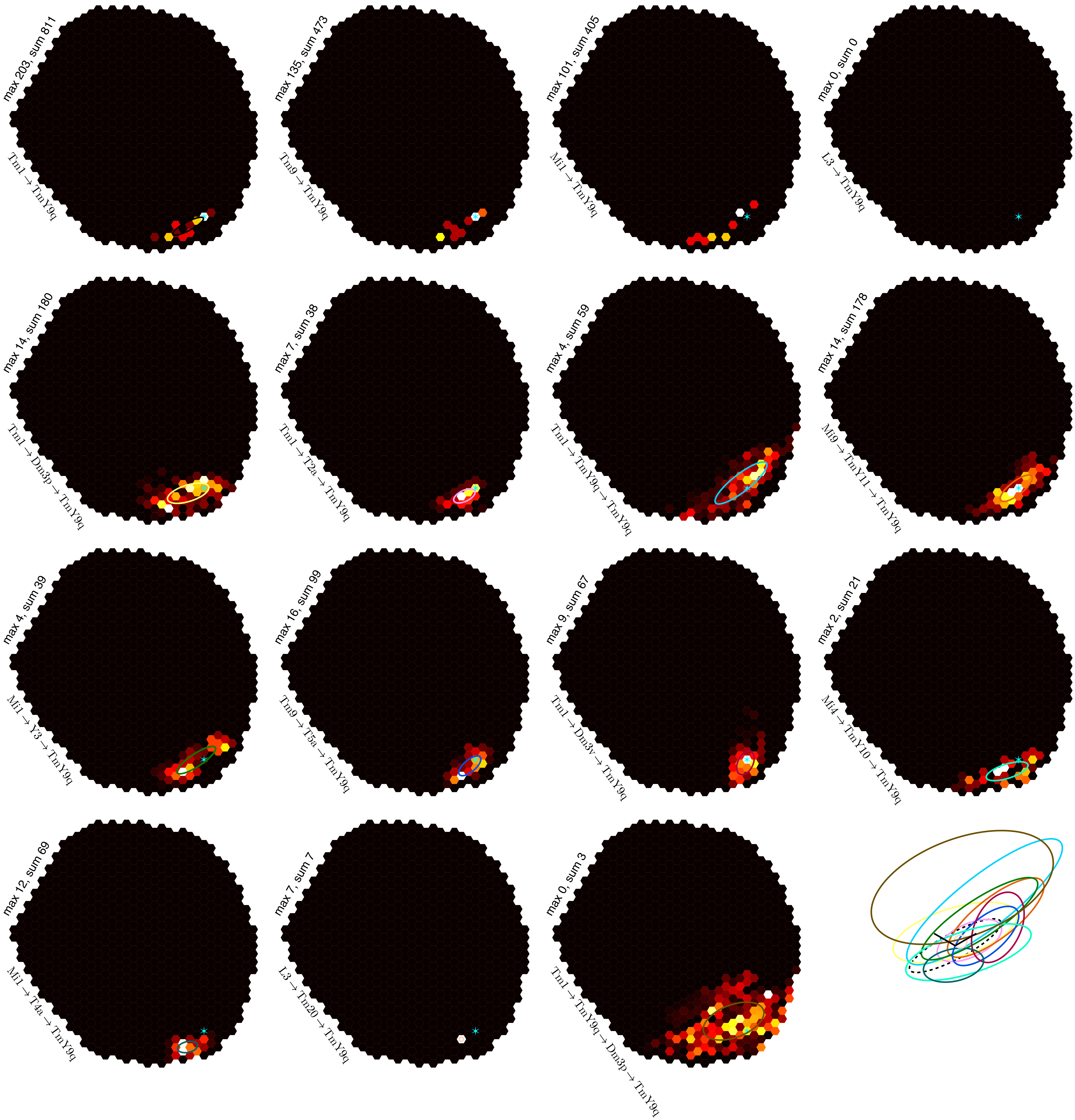

max 203, sum 811
Tm1 → TmY9q
max 135, sum 473
Tm9 → TmY9q
max 101, sum 405
Mi1 → TmY9q
max 0, sum 0
L3 → TmY9q
max 14, sum 180
Tm1 → Dm3p → TmY9q
max 7, sum 38
Tm1 → T2a → TmY9q
max 4, sum 59
Tm1 → TmY9q → TmY9q
max 14, sum 178
Mi9 → TmY11 → TmY9q
max 4, sum 39
Mi1 → Y3 → TmY9q
max 16, sum 99
Tm9 → T5a → TmY9q
max 9, sum 67
Tm1 → Dm3v → TmY9q
max 2, sum 21
Mi4 → TmY10 → TmY9q
max 12, sum 69
Mi1 → T4a → TmY9q
max 7, sum 7
L3 → Tm20 → TmY9q
max 0, sum 3
Tm1 → TmY9q → Dm3p → TmY9q
